# Three-component reaction between isatoic anhydride, amine and meth­yl-subs­tituted furyl­acryl­alde­hydes: crystal structures of 3-benzyl-2-[(*E*)-2-(5-methylfuran-2-yl)vin­yl]-2,3-di­hydro­quinazolin-4(1*H*)-one, 3-benzyl-2-[(*E*)-2-(furan-2-yl)-1-methyl­vin­yl]-2,3-di­hydro­quinazolin-4(1*H*)-one and 3-(furan-2-ylmeth­yl)-2-[(*E*)-2-(furan-2-yl)-1-methyl­vin­yl]-2,3-di­hydro­quinazolin-4(1*H*)-one

**DOI:** 10.1107/S2056989018009982

**Published:** 2018-07-13

**Authors:** Vladimir P. Zaytsev, Elena A. Sorokina, Elisaveta A. Kvyatkovskaya, Flavien A. A. Toze, Shashank N. Mhaldar, Pavel V. Dorovatovskii, Victor N. Khrustalev

**Affiliations:** a Organic Chemistry Department, Peoples’ Friendship University of Russia (RUDN University), 6 Miklukho-Maklay St., Moscow 117198, Russian Federation; b Department of Chemistry, Faculty of Sciences, University of Douala, PO Box 24157, Douala, Republic of , Cameroon; c Department of Chemistry, Goa University, Taleigao Plateau, Goa 403 206, India; d National Research Centre "Kurchatov Institute", 1 Acad. Kurchatov Sq., Moscow 123182, Russian Federation; e Inorganic Chemistry Department, Peoples’ Friendship University of Russia (RUDN University), 6 Miklukho-Maklay St., Moscow 117198, Russian Federation

**Keywords:** furans, quinazolinones, acid anhydrides, three-component reaction, crystal structure, synchrotron radiation

## Abstract

Compounds (I), C_22_H_20_N_2_O_2_, (II), C_22_H_20_N_2_O_2_ and (III), C_20_H_18_N_2_O_3_ are the products of three-component reactions between isatoic anhydride, the corresponding amine and 3-(5-methylfuran-2-yl)- or (furan-2-yl)-2-methyl­acryl­aldehyde. Compound (I) crystallizes in the monoclinic space group *P*2_1_/*n*, while compounds (II) and (III) are isostructural and crystallize in the ortho­rhom­bic space group *Pbca*. The tetra­hydro­pyrimidine ring in (I)–(III) adopts a *sofa* conformation. The NH nitro­gen atom has a trigonal–pyramidal geometry, whereas the N(*R*) nitro­gen atom is flattened. The furyl-vinyl substituents in (I)–(III) are practically planar and have an *E* configuration at the C=C double bond. In (I), this bulky fragment occupies the axial position at the quaternary carbon atom of the tetra­hydro­pyrimidine ring, whereas in (II) and (III) it is equatorially disposed. In the crystal of (I), mol­ecules form hydrogen-bonded chains propagating along [001] by strong inter­molecular N—H⋯O hydrogen bonds. The chains are packed in stacks along the *a-*axis direction. In the crystals of (II) and (III), mol­ecules also form hydrogen-bonded chains propagating along [100] by strong inter­molecular N—H⋯O hydrogen bonds. However, despite the fact that compounds (II) and (III) are isostructural, steric differences between the phenyl and furyl substituents result in chains with different geometries. Thus in the crystal of (II) the chains have a zigzag-like structure, whereas in the crystal of (III), they are almost linear. In both (II) and (III), the hydrogen-bonded chains are further packed in stacks along the *b*-axis direction.

## Abstract 2

Three 3-aryl­methyl-2-[(*E*)-2-(furan-2-yl)vin­yl]-2,3-di­hydro­quinazolin-4-ones – the products of a three-component reaction between isatoic anhydride, amine and furyl-2-methyl­acryl­aldehyde were studied by X-ray diffraction.

## Chemical context   

3-Aryl- and 3-hetaryl-substituted allyl­amines and allylic alcohols are readily available and are common starting materials for the synthesis of complex cyclic systems with useful properties (Frackenpohl *et al.*, 2016 ▸; Celltech R&D Ltd, 2004 ▸).

**Figure 1:**
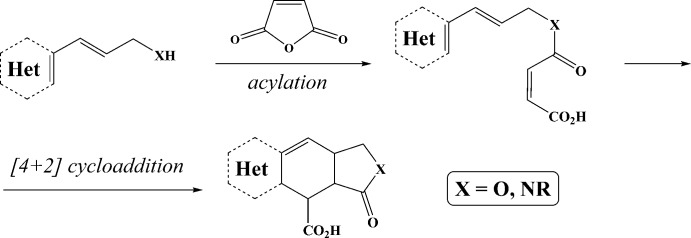
One of the synthetic pathways for the exploration of 3-substituted allyl­amines and allylic alcohols.

As depicted in Fig. 1 ▸, these substances most often undergo an *N*-acyl­ation reaction with unsaturated anhydrides or acyl chlorides to trigger the subsequent intra­molecular Diels–Alder cyclization. As a result, this sequence gives functionalized two- or three-membered heterocycles. A wide range of dienes (Tomberg *et al.*, 2015 ▸; Magedov *et al.*, 2012 ▸; Slauson *et al.*, 2015 ▸; Sun *et al.*, 2000 ▸), arenes (Hu *et al.*, 2010 ▸; Sun *et al.*, 2000 ▸; Yamazaki *et al.*, 2016 ▸; Kocsis *et al.*, 2015 ▸) and various heterocycles (Lu *et al.*, 2005 ▸; Kim *et al.*, 2014 ▸; He *et al.*, 2011 ▸) can be applied in this transformation.

Until now, only one example of the synthesis of 3-(fur­yl)allyl­amines linked to a quinazoline fragment has been described in literature (Zaytsev *et al.*, 2015 ▸). 2-Vinyl­furylquinazolinones containing no methyl groups were obtained by a three-component reaction between isatoic anhydride, a primary amine, and furylacrolein. Some further transformation of these quinazolinones has been demonstrated.

This communication pursues the aim of acquiring structural information about 2-vinyl­furylquinazolinones bearing a methyl group on the furan ring or at the double bond of the allyl­amine fragment, with the aim of further elucidating all aspects of its inter­action with α,β-unsaturated acid anhydrides.
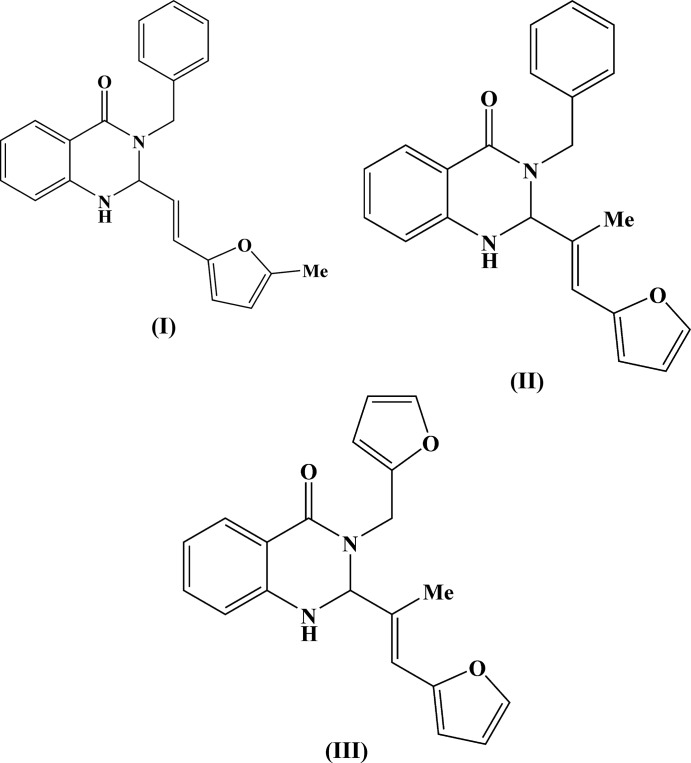


## Structural commentary   

**Figure 2:**
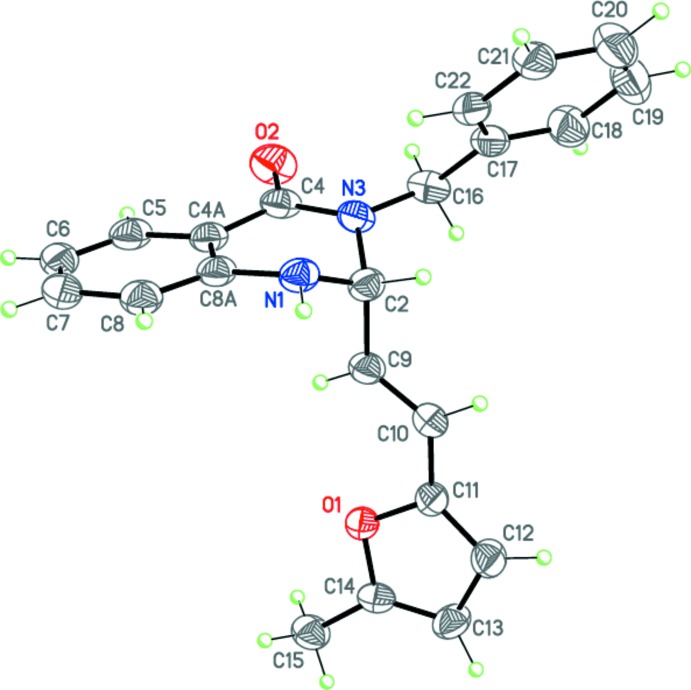
Mol­ecular structure of (I). Displacement ellipsoids are shown at the 50% probability level. H atoms are presented as small spheres of arbitrary radius.

Compounds (I), C_22_H_20_N_2_O_2_, (II), C_22_H_20_N_2_O_2_ and (III), C_20_H_18_N_2_O_3_ (Figs. 2 ▸–4 ▸ ▸) are the products of three-component reactions between isatoic anhydride, the corresponding amine and 3-(5-methylfuran-2-yl)- or (furan-2-yl)-2-methyl­acryl­aldehyde. Compound (I) crystallizes in the monoclinic space group *P*2_1_/*n*, while compounds (II) and (III) are isostructural and crystallize in the ortho­rhom­bic space group *Pbca*.

The tetra­hydro­pyrimidine ring in (I)–(III) adopts a *sofa* conformation, with the C2 carbon atom deviating from the mean plane of the other atoms of the ring by 0.639 (2), 0.476 (3) and 0.465 (3) Å, respectively. The nitro­gen atom N1 has a trigonal–pyramidal geometry [the sums of the bond angles are 345, 348 and 350° for (I)–(III), respectively], whereas the nitro­gen atom N3 is flattened [the sums of the bond angles are 357.3, 356.2 and 356.8° for (I)–(III), respectively]. The furyl-vinyl substituents in (I)–(III) are practically planar and have an *E* configuration at the C9=C10 double bond. Inter­estingly, in (I) this bulky fragment occupies the axial position at the quaternary C2 carbon atom of the tetra­hydro­pyrimidine ring, whereas in (II) and (III) it is equatorially disposed. Apparently, this may be explained by the different directions of the three-component reactions.

The mol­ecules of (I)–(III) possess an asymmetric center at the C2 carbon atom. The crystals of (I)–(III) are racemates.

## Supra­molecular features   

**Figure 5:**
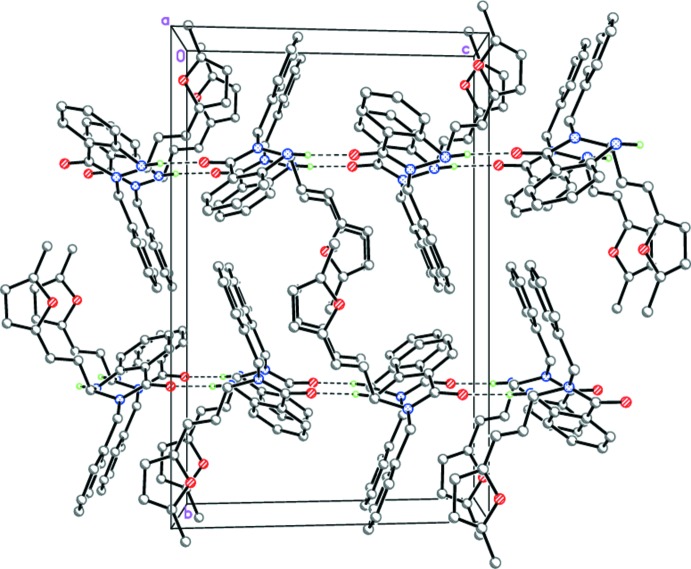
Crystal structure of (I) illustrating the N—H⋯O hydrogen-bonded chains (dashed lines) propagating along [001].

In the crystal of (I), mol­ecules form infinite hydrogen-bonded chains propagating along [001] by strong inter­molecular N1—H1⋯O2^i^ hydrogen bonds (Table 1 ▸, Fig. 5 ▸). Neighboring mol­ecules within the chains are rotated by 180° relative to each other. The chains are packed in stacks along the *a*-axis direction (Fig. 5 ▸).

**Figure 6:**
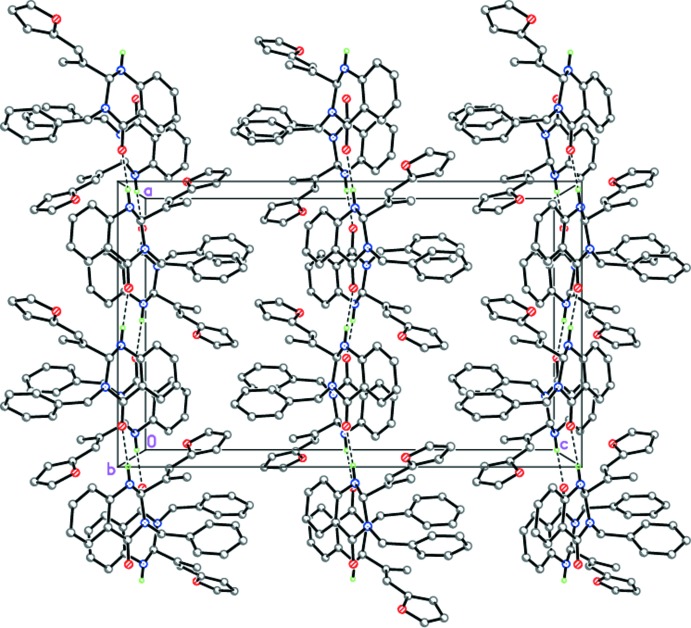
Crystal structure of (II) illustrating the zigzag N—H⋯O hydrogen-bonded chains (dashed lines) propagating along [100].

**Figure 7:**
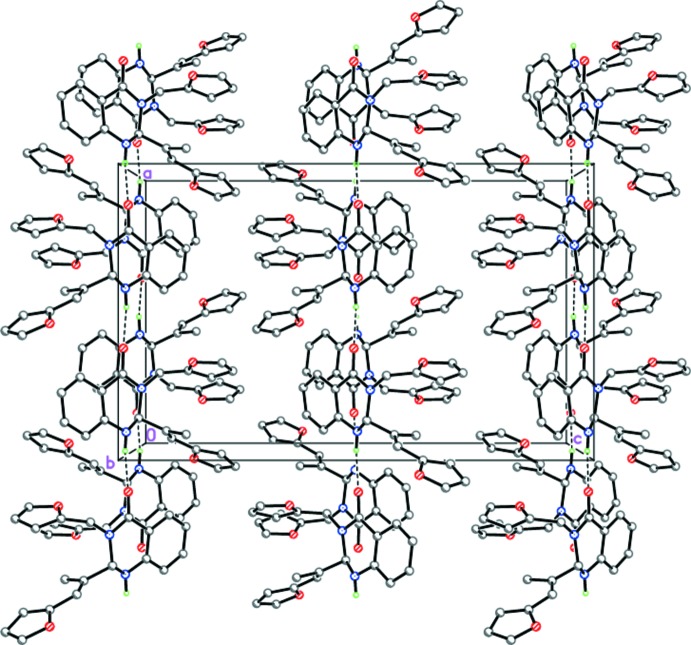
Crystal structure of (III) illustrating the almost linear N—H⋯O hydrogen-bonded chains (dashed lines) propagating along [100].

In the crystals of (II) and (III), mol­ecules also form infinite hydrogen-bonded chains propagating along [100] by strong inter­molecular N1—H1⋯O2^i^ (Table 2 ▸, Fig. 6 ▸) and N1—H1⋯O3^i^ (Table 3 ▸, Fig. 7 ▸) hydrogen bonds, respectively, with neighboring mol­ecules rotated by 180° relative to each other. However, despite the fact that compounds (II) and (III) are isostructural, steric differences between the phenyl and furyl substituents result in chains with different geometries. Thus, in the crystal of (II) the chains have a zigzag-like structure (Fig. 6 ▸), whereas in the crystal of (III) they are almost linear (Fig. 7 ▸). In both (II) and (III), the hydrogen-bonded chains are further packed in stacks along the *b*-axis direction (Figs. 6 ▸ and 7 ▸).

## Synthesis and crystallization   

3-Aryl­methyl-2-[(*E*)-2-(furan-2-yl)vin­yl]-2,3-di­hydro­quin­azolin-4-ones (I)–(III) were synthesized using a method similar to the recently described procedure (Zaytsev *et al.*, 2017 ▸).

**Figure 8:**
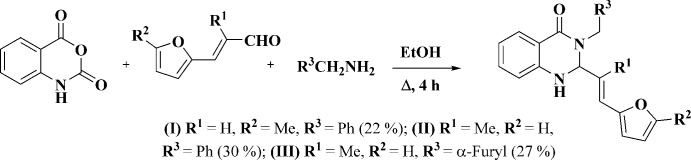
Synthesis of (I)–(III) by the three-component reaction between isatoic anhydride, the corresponding amine and 3-(5-methylfuran-2-yl)- or (furan-2-yl)-2-methyl­acryl­aldehyde.

**General procedure.** *p*-TsOH (0.79 g, 4.6 mmol) was added to a mixture of isatoic anhydride (1.5 g, 9.2 mmol), corres­ponding amine (11.0 mmol), and 3-(5-methylfuran-2-yl)- or (furan-2-yl)-2-methyl­acryl­aldehyde (9.2 mmol) in EtOH (50 mL) (Fig. 8 ▸). The reaction mixture was heated under reflux for 4 h. The progress of the reaction was monitored by TLC. When the reaction was complete, the mixture was diluted with H_2_O (100 mL) and extracted with EtOAc (3×50 mL). The organic layers were combined, dried (MgSO_4_), concentrated *in vacuo* and the residue was purified by column chromatography (3×20 cm) on SiO_2_ using hexane and then EtOAc/hexane (1/10→1/5) mixtures as eluent. The resulting product was recrystallized from a mixture hexa­ne–EtOAc to afford analytically pure samples of the target products.

**3-Benzyl-2-[(** ***E*** **)-2-(5-methylfuran-2-yl)vin­yl]-2,3-di­hydro­quin­azolin-4(1** ***H*** **)-one (I)**. Colourless needles, yield 0.7 g (22%), m.p. 430.1–432.1 K. IR (KBr), ν (cm^−1^): 3272, 1632, 1611. ^1^H NMR (CDCl_3_, 400 MHz, 301 K): δ = 2.25 (*s*, 3H, CH_3_), 3.86 (*d*, 1H, CH_2_—N, *J* = 15.1), 4.34 (*br s*, 1H, NH), 4.97 (*dd*, 1H, H2, *J* = 3.2, *J* = 4.6), 5.63 (*d*, 1H, CH_2_—N, *J* = 15.1), 5.95 (*dd*, 1H, H4, furyl, *J* = 0.9, *J* = 3.2), 6.15–6.20 (*m*, 2H, –CH=CH–, H3, fur­yl), 6.59 (*d*, 1H, H8, *J* = 7.8), 6.87 (*br t*, 1H, H6, *J* = 7.8), 7.27–7.34 (*m*, 7H, HAr, –CH=CH–), 7.99 (*dd*, 1H, H5, *J* = 1.4, *J* = 7.8). ^13^C NMR (CDCl_3_, 150.9 MHz, 301 K): δ = 13.8 (CH_3_), 46.6 (CH_2_–N), 70.1 (C2), 107.8, 111.4, 114.8, 115.8, 119.3, 121.5, 121.8, 127.6, 128.1, 128.8, 128.9, 133.6, 137.1, 145.4, 149.6, 153.1 (CAr, –CH=CH–), 162.9 (NCO). MS (EI, 70 eV): *m*/*z* = 344 [*M*]^+^ (2), 251 (16), 209 (14), 104 (10), 91 (100), 77 (20), 65 (27), 43 (24).

**3-Benzyl-2-[(** ***E*** **)-2-(furan-2-yl)-1-methyl­vin­yl]-2,3-di­hydro­quinazolin-4(1** ***H*** **)-one (II)**. Colourless plates, yield 0.95 g (30%), m.p. 405.1–406.1 K. IR (KBr), ν (cm^−1^): 3294, 1630. ^1^H NMR (CDCl_3_, 400 MHz, 301 K): δ = 1.96 (*s*, 3H, CH_3_), 3.77 (*d*, 1H, CH_2_—N, *J* = 15.1), 4.36 (*br s*, 1H, NH), 5.12 (*br s*, 1H, H2), 5.63 (*d*, 1H, CH_2_—N, *J* = 15.1), 6.11 (*s*, 1H, –C=CH–), 6.31 (*d*, 1H, H3, furyl, *J* = 3.2), 6.41 (*dd*, 1H, H4, furyl, *J* = 1.8, *J* = 3.2), 6.51 (*d*, 1H, H8, *J* = 7.8), 6.78 (*t*, 1H, H6, *J* = 7.8), 7.21–7.31 (*m*, 6H, HAr), 7.41 (*br d*, 1H, H5, furyl, *J* = 1.8), 7.94 (*dd*, 1H, H5, *J* = 1.4, *J* = 7.8). ^13^C NMR (CDCl_3_, 150.9 MHz, 301 K): δ = 13.6 (CH_3_), 46.5 (CH_2_—N), 75.1 (C2), 111.0, 111.5, 113.6, 114.4, 117.4, 118.6, 127.6, 128.2, 128.7, 128.8, 133.6, 133.8, 136.9, 142.3, 145.7, 151.8 (CAr, –C=CH–), 163.0 (NCO). MS (EI, 70 eV): *m*/*z* = 344 [*M*]^+^ (4), 237 (55), 207 (14), 167 (5), 91 (100), 77 (19), 65 (11), 44 (8).

**3-(2-Furylmeth­yl)-2-[(** ***E*** **)-2-(furan-2-yl)-1-methyl­vin­yl]-2,3-di­hydro­quinazolin-4(1** ***H*** **)-one (III).** Yellow plates, yield 0.83 g (27%), m.p. 380.1–381.1 K (hexa­ne–EtOAc). IR (KBr), ν (cm^−1^): 3308, 1632. ^1^H NMR (CDCl_3_, 400 MHz, 301 K): δ = 1.99 (*s*, 3H, CH_3_), 3.93 (*d*, 1H, CH_2_—N, *J* = 15.4), 4.22 (*br s*, 1H, NH), 5.32 (*br s*, 1H, H2), 5.39 (*d*, 1H, CH_2_—N, *J* = 15.4), 6.26 (*s*, 1H, –C=CH–), 6.28 (*br d*, 1H, H3, furyl, *J* = 3.3), 6.30 (*dd*, 1H, H4, furyl, *J* = 1.7, *J* = 3.3), 6.35 (*br d*, 1H, H3, furyl, *J* = 3.3), 6.42 (*dd*, 1H, H4, furyl, *J* = 1.7, *J* = 3.3), 6.51 (*d*, 1H, H8, *J* = 7.7), 6.78 (*t*, 1H, H6, *J* = 7.7), 7.23 (*dt*, 1H, H7, *J* = 1.1, *J* = 7.7), 7.34 (*br d*, 1H, H5, furyl, *J* = 1.7), 7.42 (*br d*, 1H, H5, furyl, *J* = 1.7), 7.92 (*dd*, 1H, H5, *J* = 1.1, *J* = 7.7). ^13^C NMR (CDCl_3_, 150.9 MHz, 301 K): δ = 13.4 (CH_3_), 39.7 (CH_2_—N), 75.8 (C2), 109.0, 110.5, 111.0, 111.5, 113.6, 114.4, 117.9, 118.6, 128.7, 133.4, 133.8, 142.3, 142.4, 145.7, 150.5, 151.8 (CAr, –C=CH–), 162.9 (NCO). MS (EI, 70 eV): *m*/*z* = 334 [*M*]^+^ (16), 227 (24), 224 (10), 81 (100), 77 (14), 53 (22).

## Refinement   

Crystal data, data collection and structure refinement details are summarized in Table 4 ▸. X-ray diffraction studies were carried out on the ‘Belok’ beamline of the National Research Center ‘Kurchatov Institute’ (Moscow, Russian Federation) using a Rayonix SX165 CCD detector. A total of 360 images for each compound was collected using an oscillation range of 1.0° (φ scan mode, two different crystal orientations) and corrected for absorption using the *SCALA* program (Evans, 2006 ▸). The data were indexed, integrated and scaled using the utility *iMosflm* in the CCP4 programme suite (Battye *et al.*, 2011 ▸).

The hydrogen atoms of the amino groups were localized in difference-Fourier maps and refined isotropically with fixed displacement parameters [*U*_iso_(H) = 1.2*U*_eq_(N)]. The other hydrogen atoms were placed in calculated positions with C—H = 0.95–1.00 Å and refined in the riding model with fixed isotropic displacement parameters [*U*_iso_(H) = 1.5*U*_eq_(C-meth­yl) or 1.2*U*_eq_(C) for all others].

A relatively large number of reflections (a few dozen) were omitted for the following reasons: (1) In order to achieve better *I*/σ statistics for high-angle reflections, we selected a longer exposure time, which resulted in some intensity overloads in the low-angle part of the area. These corrupted intensities were excluded from final steps of the refinement. (2) In the current setup of the instrument, the low-temperature device eclipses a small region of the detector near its high-angle limit. This resulted in zero intensity for some reflections. (3) The quality of the single crystals chosen for the diffraction experiments was far from perfect. Some systematic intensity deviations can be due to extinction and defects present in the crystals.

## Supplementary Material

Crystal structure: contains datablock(s) global, I, II, III. DOI: 10.1107/S2056989018009982/yk2115sup1.cif

Structure factors: contains datablock(s) I. DOI: 10.1107/S2056989018009982/yk2115Isup2.hkl

Structure factors: contains datablock(s) II. DOI: 10.1107/S2056989018009982/yk2115IIsup3.hkl

Structure factors: contains datablock(s) III. DOI: 10.1107/S2056989018009982/yk2115IIIsup4.hkl

CCDC references: 1855145, 1855144, 1855143

Additional supporting information:  crystallographic information; 3D view; checkCIF report

## Figures and Tables

**Figure 3:**
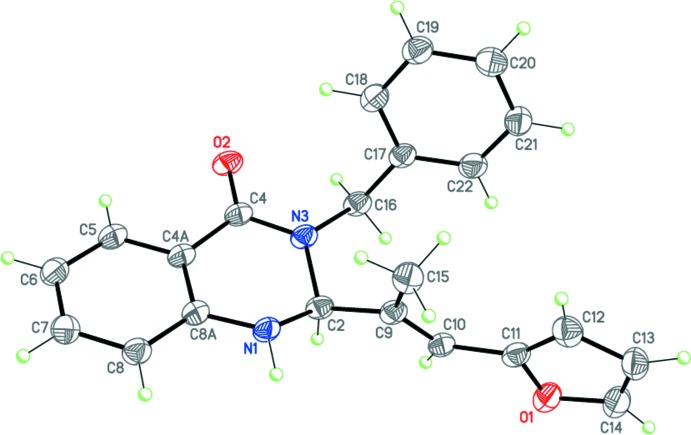
Mol­ecular structure of (II). Displacement ellipsoids are shown at the 50% probability level. H atoms are presented as small spheres of arbitrary radius.

**Figure 4:**
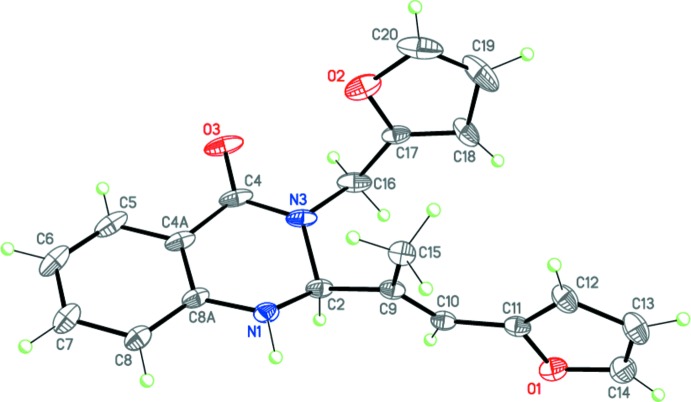
Mol­ecular structure of (III). Displacement ellipsoids are shown at the 50% probability level. H atoms are presented as small spheres of arbitrary radius.

**Table 1:** Hydrogen-bond geometry (Å, °) for (I)

*D*—H⋯*A*	*D*—H	H⋯*A*	*D*⋯*A*	*D*—H⋯*A*
N1—H1⋯O2^i^	0.92 (2)	1.92 (2)	2.817 (2)	164.4 (19)

Symmetry code: (i) 
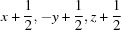
.

**Table 2:** Hydrogen-bond geometry (Å, °) for (II)

*D*—H⋯*A*	*D*—H	H⋯*A*	*D*⋯*A*	*D*—H⋯*A*
N1—H1⋯O2^i^	0.90 (3)	2.07 (3)	2.971 (3)	174 (2)

Symmetry code: (i) 
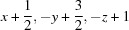
.

**Table 3:** Hydrogen-bond geometry (Å, °) for (III)

*D*—H⋯*A*	*D*—H	H⋯*A*	*D*⋯*A*	*D*—H⋯*A*
N1—H1⋯O3^i^	0.92 (4)	2.04 (4)	2.949 (4)	169 (3)

Symmetry code: (i) 
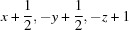
.

**Table 4:** Experimental details

	(I)	(II)	(III)
Crystal data			
Chemical formula	C_22_H_20_N_2_O_2_	C_22_H_20_N_2_O_2_	C_20_H_18_N_2_O_3_
*M*_r_	344.40	344.40	334.36
Crystal system, space group	Monoclinic, *P*2_1_/*n*	Orthorhombic, *P* *b* *c* *a*	Orthorhombic, *P* *b* *c* *a*
Temperature (K)	100	100	100
*a*, *b*, *c* (Å)	7.9416 (16), 19.202 (4), 12.497 (3)	13.921 (3), 11.296 (2), 22.623 (5)	13.928 (3), 10.684 (2), 22.368 (5)
α, β, γ (°)	90, 99.663 (3), 90	90, 90, 90	90, 90, 90
*V* (Å^3^)	1878.7 (7)	3557.5 (13)	3328.5 (12)
*Z*	4	8	8
Radiation type	Synchrotron, λ = 0.96990 Å	Synchrotron, λ = 0.96990 Å	Synchrotron, λ = 0.96990 Å
μ (mm^−1^)	0.16	0.17	0.19
Crystal size (mm)	0.25 × 0.08 × 0.03	0.20 × 0.15 × 0.01	0.30 × 0.30 × 0.07

Data collection			
Diffractometer	Rayonix SX165 CCD	Rayonix SX165 CCD	Rayonix SX165 CCD
Absorption correction	Multi-scan (*SCALA*; Evans, 2006 ▸)	Multi-scan (*SCALA*; Evans, 2006 ▸)	Multi-scan (*SCALA*; Evans, 2006 ▸)
*T*_min_, *T*_max_	0.950, 0.990	0.960, 0.990	0.940, 0.980
No. of measured, independent and observed [*I* > 2σ(*I*)] reflections	20568, 3781, 2264	18942, 3764, 2411	27461, 3460, 2414
*R*_int_	0.080	0.070	0.097
(sin θ/λ)_max_ (Å^−1^)	0.640	0.640	0.641

Refinement			
*R*[*F* ^2^ > 2σ(*F* ^2^)], *wR*(*F* ^2^), *S*	0.097, 0.218, 1.00	0.074, 0.190, 1.05	0.089, 0.224, 1.05
No. of reflections	3781	3764	3460
No. of parameters	240	240	231
H-atom treatment	H atoms treated by a mixture of independent and constrained refinement	H atoms treated by a mixture of independent and constrained refinement	H atoms treated by a mixture of independent and constrained refinement
Δρ_max_, Δρ_min_ (e Å^−3^)	0.37, −0.31	0.30, −0.28	0.42, −0.57

Computer programs: *Marccd* (Doyle, 2011 ▸), *iMosflm* (Battye *et al.*, 2011 ▸), *SHELXT* (Sheldrick, 2015*a* ▸) and *SHELXL* (Sheldrick, 2015*b* ▸), *SHELXTL* (Sheldrick, 2008 ▸).

## supplementary crystallographic information

### 3-Benzyl-2-[(E)-2-(5-methylfuran-2-yl)vinyl]-2,3-dihydroquinazolin-4(1H)-one (I) Crystal data

**Table:**

C_22_H_20_N_2_O_2_	*F*(000) = 728
*M**_r_* = 344.40	*D*_x_ = 1.218 Mg m^−^^3^
Monoclinic, *P*2_1_/*n*	Synchrotron radiation, λ = 0.96990 Å
*a* = 7.9416 (16) Å	Cell parameters from 500 reflections
*b* = 19.202 (4) Å	θ = 3.5–35.0°
*c* = 12.497 (3) Å	µ = 0.16 mm^−^^1^
β = 99.663 (3)°	*T* = 100 K
*V* = 1878.7 (7) Å^3^	Needle, colourless
*Z* = 4	0.25 × 0.08 × 0.03 mm

### 3-Benzyl-2-[(E)-2-(5-methylfuran-2-yl)vinyl]-2,3-dihydroquinazolin-4(1H)-one (I) Data collection

**Table:**

Rayonix SX165 CCD diffractometer	2264 reflections with *I* > 2σ(*I*)
/f scan	*R*_int_ = 0.080
Absorption correction: multi-scan (*SCALA*; Evans, 2006)	θ_max_ = 38.4°, θ_min_ = 3.7°
*T*_min_ = 0.950, *T*_max_ = 0.990	*h* = −8→9
20568 measured reflections	*k* = −24→24
3781 independent reflections	*l* = −15→15

### 3-Benzyl-2-[(E)-2-(5-methylfuran-2-yl)vinyl]-2,3-dihydroquinazolin-4(1H)-one (I) Refinement

**Table:**

Refinement on *F*^2^	Secondary atom site location: difference Fourier map
Least-squares matrix: full	Hydrogen site location: mixed
*R*[*F*^2^ > 2σ(*F*^2^)] = 0.097	H atoms treated by a mixture of independent and constrained refinement
*wR*(*F*^2^) = 0.218	*w* = 1/[σ^2^(*F*_o_^2^)] where *P* = (*F*_o_^2^ + 2*F*_c_^2^)/3
*S* = 1.00	(Δ/σ)_max_ < 0.001
3781 reflections	Δρ_max_ = 0.37 e Å^−^^3^
240 parameters	Δρ_min_ = −0.31 e Å^−^^3^
0 restraints	Extinction correction: SHELXL, Fc^*^=kFc[1+0.001xFc^2^λ^3^/sin(2θ)]^-1/4^
Primary atom site location: difference Fourier map	Extinction coefficient: 0.085 (8)

### 3-Benzyl-2-[(E)-2-(5-methylfuran-2-yl)vinyl]-2,3-dihydroquinazolin-4(1H)-one (I) Special details

**Table:**

Geometry. All esds (except the esd in the dihedral angle between two l.s. planes) are estimated using the full covariance matrix. The cell esds are taken into account individually in the estimation of esds in distances, angles and torsion angles; correlations between esds in cell parameters are only used when they are defined by crystal symmetry. An approximate (isotropic) treatment of cell esds is used for estimating esds involving l.s. planes.

### 3-Benzyl-2-[(E)-2-(5-methylfuran-2-yl)vinyl]-2,3-dihydroquinazolin-4(1H)-one (I) Fractional atomic coordinates and isotropic or equivalent isotropic displacement parameters (Å^2^)

**Table:**

	*x*	*y*	*z*	*U*_iso_*/*U*_eq_	
O1	0.28682 (18)	0.44379 (6)	0.47756 (11)	0.0354 (5)	
O2	0.2968 (2)	0.25305 (7)	0.07159 (12)	0.0523 (5)	
N1	0.6282 (3)	0.24206 (7)	0.35460 (14)	0.0350 (5)	
H1	0.701 (3)	0.2458 (8)	0.4204 (18)	0.042*	
C2	0.4453 (3)	0.24277 (9)	0.35967 (16)	0.0346 (6)	
H2	0.4234	0.2060	0.4124	0.042*	
N3	0.3532 (2)	0.22258 (8)	0.25120 (12)	0.0351 (5)	
C4	0.3922 (3)	0.25582 (9)	0.16113 (18)	0.0378 (6)	
C4A	0.5591 (3)	0.29244 (9)	0.17600 (15)	0.0347 (6)	
C5	0.6075 (3)	0.33384 (9)	0.09282 (15)	0.0428 (7)	
H5	0.5278	0.3418	0.0283	0.051*	
C6	0.7685 (3)	0.36302 (10)	0.10347 (17)	0.0440 (7)	
H6	0.7987	0.3912	0.0472	0.053*	
C7	0.8863 (3)	0.35072 (9)	0.19744 (18)	0.0437 (6)	
H7	0.9980	0.3698	0.2039	0.052*	
C8	0.8432 (3)	0.31105 (9)	0.28204 (17)	0.0398 (6)	
H8	0.9249	0.3032	0.3457	0.048*	
C8A	0.6779 (3)	0.28254 (9)	0.27292 (15)	0.0348 (6)	
C9	0.3811 (3)	0.31155 (9)	0.39732 (15)	0.0353 (6)	
H9	0.3892	0.3518	0.3543	0.042*	
C10	0.3141 (3)	0.31829 (10)	0.48751 (16)	0.0355 (6)	
H10	0.3002	0.2769	0.5267	0.043*	
C11	0.2603 (3)	0.38264 (9)	0.53159 (16)	0.0352 (6)	
C12	0.1939 (3)	0.39832 (9)	0.62208 (16)	0.0415 (6)	
H12	0.1636	0.3661	0.6733	0.050*	
C13	0.1783 (3)	0.47279 (10)	0.62563 (16)	0.0417 (6)	
H13	0.1359	0.4991	0.6798	0.050*	
C14	0.2355 (3)	0.49853 (9)	0.53705 (17)	0.0355 (6)	
C15	0.2558 (3)	0.56945 (8)	0.49272 (18)	0.0433 (6)	
H15A	0.3767	0.5780	0.4903	0.065*	
H15B	0.2143	0.6042	0.5395	0.065*	
H15C	0.1897	0.5728	0.4193	0.065*	
C16	0.1931 (3)	0.18476 (10)	0.24752 (16)	0.0416 (6)	
H16A	0.1365	0.1808	0.1709	0.050*	
H16B	0.1170	0.2122	0.2866	0.050*	
C17	0.2130 (3)	0.11236 (10)	0.29657 (16)	0.0377 (6)	
C18	0.0692 (3)	0.07756 (11)	0.31936 (18)	0.0490 (7)	
H18	−0.0384	0.1003	0.3063	0.059*	
C19	0.0799 (4)	0.00989 (12)	0.3611 (2)	0.0599 (8)	
H19	−0.0201	−0.0134	0.3744	0.072*	
C20	0.2384 (4)	−0.02327 (12)	0.38311 (18)	0.0601 (8)	
H20	0.2472	−0.0691	0.4121	0.072*	
C21	0.3821 (4)	0.01103 (10)	0.36237 (16)	0.0485 (7)	
H21	0.4904	−0.0112	0.3779	0.058*	
C22	0.3698 (3)	0.07805 (10)	0.31880 (16)	0.0410 (7)	
H22	0.4697	0.1007	0.3040	0.049*	

### 3-Benzyl-2-[(E)-2-(5-methylfuran-2-yl)vinyl]-2,3-dihydroquinazolin-4(1H)-one (I) Atomic displacement parameters (Å^2^)

**Table:**

	*U*^11^	*U*^22^	*U*^33^	*U*^12^	*U*^13^	*U*^23^
O1	0.0423 (11)	0.0291 (8)	0.0371 (9)	−0.0006 (6)	0.0135 (7)	−0.0021 (5)
O2	0.0677 (14)	0.0512 (10)	0.0321 (10)	−0.0035 (8)	−0.0082 (9)	0.0004 (6)
N1	0.0476 (15)	0.0303 (10)	0.0265 (10)	0.0035 (8)	0.0042 (9)	0.0001 (7)
C2	0.0437 (17)	0.0324 (11)	0.0278 (12)	0.0006 (9)	0.0066 (11)	−0.0010 (8)
N3	0.0454 (14)	0.0274 (9)	0.0314 (10)	−0.0021 (8)	0.0028 (9)	−0.0021 (7)
C4	0.0564 (19)	0.0256 (10)	0.0298 (13)	0.0040 (10)	0.0024 (12)	−0.0020 (9)
C4A	0.0507 (17)	0.0254 (10)	0.0286 (12)	0.0041 (10)	0.0088 (11)	−0.0025 (8)
C5	0.069 (2)	0.0277 (11)	0.0327 (13)	0.0061 (12)	0.0115 (12)	−0.0006 (8)
C6	0.069 (2)	0.0262 (11)	0.0415 (14)	−0.0023 (12)	0.0215 (13)	0.0001 (9)
C7	0.0540 (18)	0.0276 (11)	0.0528 (15)	−0.0001 (10)	0.0192 (14)	−0.0041 (10)
C8	0.0499 (18)	0.0288 (11)	0.0418 (13)	0.0049 (10)	0.0110 (12)	−0.0034 (9)
C8A	0.0512 (17)	0.0240 (10)	0.0302 (12)	0.0036 (10)	0.0095 (11)	−0.0020 (8)
C9	0.0477 (16)	0.0259 (10)	0.0324 (12)	−0.0009 (9)	0.0069 (11)	−0.0011 (8)
C10	0.0397 (16)	0.0287 (11)	0.0382 (12)	−0.0031 (9)	0.0076 (11)	−0.0008 (8)
C11	0.0428 (16)	0.0291 (11)	0.0357 (12)	−0.0006 (10)	0.0125 (11)	0.0002 (8)
C12	0.0507 (17)	0.0327 (11)	0.0438 (14)	0.0032 (10)	0.0158 (12)	0.0045 (9)
C13	0.0508 (17)	0.0380 (12)	0.0396 (13)	0.0078 (11)	0.0168 (12)	−0.0041 (9)
C14	0.0389 (15)	0.0289 (11)	0.0382 (12)	0.0021 (9)	0.0048 (11)	−0.0066 (9)
C15	0.0516 (18)	0.0313 (12)	0.0471 (14)	0.0006 (10)	0.0084 (12)	−0.0017 (9)
C16	0.0421 (17)	0.0343 (12)	0.0463 (14)	0.0005 (11)	0.0016 (11)	−0.0091 (9)
C17	0.0490 (17)	0.0306 (11)	0.0336 (12)	−0.0009 (11)	0.0070 (11)	−0.0057 (9)
C18	0.0476 (18)	0.0475 (14)	0.0512 (15)	−0.0068 (12)	0.0063 (12)	−0.0027 (11)
C19	0.063 (2)	0.0498 (15)	0.0672 (18)	−0.0180 (14)	0.0125 (15)	0.0054 (11)
C20	0.088 (2)	0.0374 (13)	0.0567 (16)	−0.0095 (15)	0.0158 (15)	0.0045 (11)
C21	0.071 (2)	0.0307 (12)	0.0466 (14)	0.0065 (12)	0.0193 (13)	0.0005 (10)
C22	0.0602 (19)	0.0296 (11)	0.0371 (13)	0.0012 (11)	0.0192 (12)	−0.0031 (9)

### 3-Benzyl-2-[(E)-2-(5-methylfuran-2-yl)vinyl]-2,3-dihydroquinazolin-4(1H)-one (I) Geometric parameters (Å, º)

**Table:**

O1—C14	1.388 (2)	C10—H10	0.9500
O1—C11	1.388 (2)	C11—C12	1.359 (3)
O2—C4	1.243 (2)	C12—C13	1.437 (3)
N1—C8A	1.392 (2)	C12—H12	0.9500
N1—C2	1.465 (3)	C13—C14	1.358 (3)
N1—H1	0.92 (2)	C13—H13	0.9500
C2—N3	1.480 (3)	C14—C15	1.489 (2)
C2—C9	1.518 (2)	C15—H15A	0.9800
C2—H2	1.0000	C15—H15B	0.9800
N3—C4	1.374 (2)	C15—H15C	0.9800
N3—C16	1.459 (3)	C16—C17	1.517 (3)
C4—C4A	1.485 (3)	C16—H16A	0.9900
C4A—C5	1.412 (2)	C16—H16B	0.9900
C4A—C8A	1.417 (3)	C17—C18	1.393 (3)
C5—C6	1.382 (3)	C17—C22	1.394 (3)
C5—H5	0.9500	C18—C19	1.397 (3)
C6—C7	1.393 (3)	C18—H18	0.9500
C6—H6	0.9500	C19—C20	1.396 (4)
C7—C8	1.392 (3)	C19—H19	0.9500
C7—H7	0.9500	C20—C21	1.380 (3)
C8—C8A	1.410 (3)	C20—H20	0.9500
C8—H8	0.9500	C21—C22	1.394 (2)
C9—C10	1.331 (2)	C21—H21	0.9500
C9—H9	0.9500	C22—H22	0.9500
C10—C11	1.446 (2)		
			
C14—O1—C11	107.34 (15)	C12—C11—C10	133.37 (18)
C8A—N1—C2	115.58 (17)	O1—C11—C10	117.32 (17)
C8A—N1—H1	113.4 (12)	C11—C12—C13	107.09 (16)
C2—N1—H1	116.0 (13)	C11—C12—H12	126.5
N1—C2—N3	107.40 (15)	C13—C12—H12	126.5
N1—C2—C9	114.10 (15)	C14—C13—C12	107.13 (15)
N3—C2—C9	111.83 (16)	C14—C13—H13	126.4
N1—C2—H2	107.8	C12—C13—H13	126.4
N3—C2—H2	107.8	C13—C14—O1	109.23 (15)
C9—C2—H2	107.8	C13—C14—C15	135.07 (18)
C4—N3—C16	121.37 (18)	O1—C14—C15	115.69 (17)
C4—N3—C2	118.97 (17)	C14—C15—H15A	109.5
C16—N3—C2	116.97 (15)	C14—C15—H15B	109.5
O2—C4—N3	122.1 (2)	H15A—C15—H15B	109.5
O2—C4—C4A	121.97 (19)	C14—C15—H15C	109.5
N3—C4—C4A	115.89 (19)	H15A—C15—H15C	109.5
C5—C4A—C8A	118.7 (2)	H15B—C15—H15C	109.5
C5—C4A—C4	121.5 (2)	N3—C16—C17	114.34 (19)
C8A—C4A—C4	119.71 (18)	N3—C16—H16A	108.7
C6—C5—C4A	121.3 (2)	C17—C16—H16A	108.7
C6—C5—H5	119.4	N3—C16—H16B	108.7
C4A—C5—H5	119.4	C17—C16—H16B	108.7
C5—C6—C7	119.46 (18)	H16A—C16—H16B	107.6
C5—C6—H6	120.3	C18—C17—C22	117.9 (2)
C7—C6—H6	120.3	C18—C17—C16	119.2 (2)
C8—C7—C6	121.1 (2)	C22—C17—C16	122.9 (2)
C8—C7—H7	119.4	C17—C18—C19	121.5 (2)
C6—C7—H7	119.4	C17—C18—H18	119.3
C7—C8—C8A	119.7 (2)	C19—C18—H18	119.3
C7—C8—H8	120.1	C20—C19—C18	119.6 (2)
C8A—C8—H8	120.1	C20—C19—H19	120.2
N1—C8A—C8	122.2 (2)	C18—C19—H19	120.2
N1—C8A—C4A	118.1 (2)	C21—C20—C19	119.5 (2)
C8—C8A—C4A	119.62 (18)	C21—C20—H20	120.3
C10—C9—C2	123.34 (17)	C19—C20—H20	120.3
C10—C9—H9	118.3	C20—C21—C22	120.5 (2)
C2—C9—H9	118.3	C20—C21—H21	119.7
C9—C10—C11	126.24 (18)	C22—C21—H21	119.7
C9—C10—H10	116.9	C21—C22—C17	121.1 (2)
C11—C10—H10	116.9	C21—C22—H22	119.5
C12—C11—O1	109.20 (16)	C17—C22—H22	119.5
			
C8A—N1—C2—N3	54.70 (19)	N1—C2—C9—C10	−117.6 (2)
C8A—N1—C2—C9	−69.8 (2)	N3—C2—C9—C10	120.2 (2)
N1—C2—N3—C4	−49.9 (2)	C2—C9—C10—C11	175.94 (19)
C9—C2—N3—C4	76.0 (2)	C14—O1—C11—C12	0.2 (2)
N1—C2—N3—C16	148.38 (16)	C14—O1—C11—C10	−176.56 (17)
C9—C2—N3—C16	−85.71 (19)	C9—C10—C11—C12	−178.5 (2)
C16—N3—C4—O2	−2.9 (3)	C9—C10—C11—O1	−2.7 (3)
C2—N3—C4—O2	−163.75 (17)	O1—C11—C12—C13	−0.2 (2)
C16—N3—C4—C4A	179.93 (16)	C10—C11—C12—C13	175.8 (2)
C2—N3—C4—C4A	19.1 (2)	C11—C12—C13—C14	0.1 (2)
O2—C4—C4A—C5	8.7 (3)	C12—C13—C14—O1	0.0 (2)
N3—C4—C4A—C5	−174.14 (15)	C12—C13—C14—C15	−179.3 (3)
O2—C4—C4A—C8A	−167.33 (16)	C11—O1—C14—C13	−0.1 (2)
N3—C4—C4A—C8A	9.8 (3)	C11—O1—C14—C15	179.34 (18)
C8A—C4A—C5—C6	1.6 (3)	C4—N3—C16—C17	131.92 (18)
C4—C4A—C5—C6	−174.45 (18)	C2—N3—C16—C17	−66.9 (2)
C4A—C5—C6—C7	0.8 (3)	N3—C16—C17—C18	165.99 (17)
C5—C6—C7—C8	−1.7 (3)	N3—C16—C17—C22	−15.0 (3)
C6—C7—C8—C8A	0.1 (3)	C22—C17—C18—C19	−1.3 (3)
C2—N1—C8A—C8	152.99 (17)	C16—C17—C18—C19	177.8 (2)
C2—N1—C8A—C4A	−29.7 (2)	C17—C18—C19—C20	1.6 (3)
C7—C8—C8A—N1	179.53 (15)	C18—C19—C20—C21	−0.6 (4)
C7—C8—C8A—C4A	2.3 (3)	C19—C20—C21—C22	−0.6 (3)
C5—C4A—C8A—N1	179.53 (15)	C20—C21—C22—C17	0.9 (3)
C4—C4A—C8A—N1	−4.3 (3)	C18—C17—C22—C21	0.1 (3)
C5—C4A—C8A—C8	−3.1 (3)	C16—C17—C22—C21	−178.98 (17)
C4—C4A—C8A—C8	172.99 (15)		

### 3-Benzyl-2-[(E)-2-(5-methylfuran-2-yl)vinyl]-2,3-dihydroquinazolin-4(1H)-one (I) Hydrogen-bond geometry (Å, º)

**Table:**

*D*—H···*A*	*D*—H	H···*A*	*D*···*A*	*D*—H···*A*
N1—H1···O2^i^	0.92 (2)	1.92 (2)	2.817 (2)	164.4 (19)

Symmetry code: (i) *x*+1/2, −*y*+1/2, *z*+1/2.

### 3-Benzyl-2-[(E)-2-(furan-2-yl)-1-methylvinyl]-2,3-dihydroquinazolin-4(1H)-one (II) Crystal data

**Table:**

C_22_H_20_N_2_O_2_	*D*_x_ = 1.286 Mg m^−^^3^
*M**_r_* = 344.40	Synchrotron radiation, λ = 0.96990 Å
Orthorhombic, *P**b**c**a*	Cell parameters from 500 reflections
*a* = 13.921 (3) Å	θ = 3.2–32.0°
*b* = 11.296 (2) Å	µ = 0.17 mm^−^^1^
*c* = 22.623 (5) Å	*T* = 100 K
*V* = 3557.5 (13) Å^3^	Plate, colourless
*Z* = 8	0.20 × 0.15 × 0.01 mm
*F*(000) = 1456	

### 3-Benzyl-2-[(E)-2-(furan-2-yl)-1-methylvinyl]-2,3-dihydroquinazolin-4(1H)-one (II) Data collection

**Table:**

Rayonix SX165 CCD diffractometer	2411 reflections with *I* > 2σ(*I*)
/f scan	*R*_int_ = 0.070
Absorption correction: multi-scan (*SCALA*; Evans, 2006)	θ_max_ = 38.4°, θ_min_ = 3.2°
*T*_min_ = 0.960, *T*_max_ = 0.990	*h* = −17→17
18942 measured reflections	*k* = −14→14
3764 independent reflections	*l* = −28→28

### 3-Benzyl-2-[(E)-2-(furan-2-yl)-1-methylvinyl]-2,3-dihydroquinazolin-4(1H)-one (II) Refinement

**Table:**

Refinement on *F*^2^	Secondary atom site location: difmap2
Least-squares matrix: full	Hydrogen site location: mixed
*R*[*F*^2^ > 2σ(*F*^2^)] = 0.074	H atoms treated by a mixture of independent and constrained refinement
*wR*(*F*^2^) = 0.190	*w* = 1/[σ^2^(*F*_o_^2^) + 2*P*] where *P* = (*F*_o_^2^ + 2*F*_c_^2^)/3
*S* = 1.05	(Δ/σ)_max_ < 0.001
3764 reflections	Δρ_max_ = 0.30 e Å^−^^3^
240 parameters	Δρ_min_ = −0.28 e Å^−^^3^
0 restraints	Extinction correction: SHELXL, Fc^*^=kFc[1+0.001xFc^2^λ^3^/sin(2θ)]^-1/4^
Primary atom site location: difference Fourier map	Extinction coefficient: 0.0102 (10)

### 3-Benzyl-2-[(E)-2-(furan-2-yl)-1-methylvinyl]-2,3-dihydroquinazolin-4(1H)-one (II) Special details

**Table:**

Geometry. All esds (except the esd in the dihedral angle between two l.s. planes) are estimated using the full covariance matrix. The cell esds are taken into account individually in the estimation of esds in distances, angles and torsion angles; correlations between esds in cell parameters are only used when they are defined by crystal symmetry. An approximate (isotropic) treatment of cell esds is used for estimating esds involving l.s. planes.

### 3-Benzyl-2-[(E)-2-(furan-2-yl)-1-methylvinyl]-2,3-dihydroquinazolin-4(1H)-one (II) Fractional atomic coordinates and isotropic or equivalent isotropic displacement parameters (Å^2^)

**Table:**

	*x*	*y*	*z*	*U*_iso_*/*U*_eq_	
O1	0.54343 (13)	0.33368 (16)	0.38217 (8)	0.0430 (5)	
O2	0.13105 (12)	0.75727 (16)	0.49206 (8)	0.0405 (5)	
N1	0.42208 (15)	0.77868 (19)	0.48819 (9)	0.0339 (5)	
H1	0.486 (2)	0.772 (2)	0.4927 (11)	0.041*	
C2	0.37955 (17)	0.6658 (2)	0.47071 (11)	0.0328 (6)	
H2	0.3889	0.6072	0.5033	0.039*	
N3	0.27451 (14)	0.68234 (18)	0.46041 (9)	0.0326 (5)	
C4	0.21959 (18)	0.7574 (2)	0.49452 (11)	0.0329 (6)	
C4A	0.27357 (17)	0.8430 (2)	0.53208 (11)	0.0329 (6)	
C5	0.22296 (18)	0.9208 (2)	0.56907 (11)	0.0364 (6)	
H5	0.1552	0.9134	0.5727	0.044*	
C6	0.27111 (19)	1.0090 (2)	0.60050 (11)	0.0395 (7)	
H6	0.2366	1.0619	0.6252	0.047*	
C7	0.3704 (2)	1.0183 (2)	0.59511 (11)	0.0392 (6)	
H7	0.4035	1.0785	0.6162	0.047*	
C8	0.42221 (18)	0.9407 (2)	0.55936 (11)	0.0365 (6)	
H8	0.4901	0.9476	0.5566	0.044*	
C8A	0.37381 (17)	0.8521 (2)	0.52727 (10)	0.0317 (6)	
C9	0.43039 (16)	0.6209 (2)	0.41535 (11)	0.0319 (6)	
C10	0.46061 (16)	0.5072 (2)	0.41468 (11)	0.0334 (6)	
H10	0.4491	0.4625	0.4495	0.040*	
C11	0.50873 (17)	0.4455 (2)	0.36658 (11)	0.0354 (6)	
C12	0.52915 (19)	0.4665 (2)	0.30866 (12)	0.0421 (7)	
H12	0.5136	0.5356	0.2867	0.051*	
C13	0.5792 (2)	0.3627 (3)	0.28702 (12)	0.0441 (7)	
H13	0.6028	0.3503	0.2481	0.053*	
C14	0.5856 (2)	0.2871 (3)	0.33296 (13)	0.0461 (7)	
H14	0.6156	0.2116	0.3313	0.055*	
C15	0.4437 (2)	0.7071 (2)	0.36493 (11)	0.0405 (7)	
H15A	0.5122	0.7138	0.3555	0.061*	
H15B	0.4188	0.7849	0.3765	0.061*	
H15C	0.4087	0.6786	0.3301	0.061*	
C16	0.22429 (17)	0.5853 (2)	0.42882 (11)	0.0356 (6)	
H16A	0.1591	0.5760	0.4456	0.043*	
H16B	0.2595	0.5104	0.4354	0.043*	
C17	0.21601 (17)	0.6074 (2)	0.36272 (11)	0.0337 (6)	
C18	0.16896 (19)	0.7080 (2)	0.34142 (12)	0.0387 (6)	
H18	0.1399	0.7615	0.3684	0.046*	
C19	0.1643 (2)	0.7306 (2)	0.28113 (12)	0.0406 (7)	
H19	0.1317	0.7992	0.2673	0.049*	
C20	0.2067 (2)	0.6539 (3)	0.24086 (12)	0.0428 (7)	
H20	0.2048	0.6708	0.1997	0.051*	
C21	0.2521 (2)	0.5522 (3)	0.26139 (13)	0.0461 (7)	
H21	0.2803	0.4983	0.2343	0.055*	
C22	0.25596 (19)	0.5296 (2)	0.32196 (12)	0.0421 (7)	
H22	0.2865	0.4596	0.3356	0.051*	

### 3-Benzyl-2-[(E)-2-(furan-2-yl)-1-methylvinyl]-2,3-dihydroquinazolin-4(1H)-one (II) Atomic displacement parameters (Å^2^)

**Table:**

	*U*^11^	*U*^22^	*U*^33^	*U*^12^	*U*^13^	*U*^23^
O1	0.0384 (11)	0.0384 (11)	0.0522 (12)	0.0046 (8)	0.0039 (9)	−0.0038 (9)
O2	0.0210 (9)	0.0485 (11)	0.0520 (12)	0.0021 (8)	0.0008 (8)	0.0008 (9)
N1	0.0192 (10)	0.0379 (13)	0.0445 (13)	0.0006 (9)	0.0006 (9)	−0.0058 (10)
C2	0.0225 (12)	0.0362 (14)	0.0397 (14)	−0.0014 (10)	−0.0003 (10)	0.0011 (11)
N3	0.0210 (10)	0.0353 (12)	0.0415 (12)	−0.0007 (8)	0.0012 (9)	−0.0012 (10)
C4	0.0243 (12)	0.0374 (14)	0.0370 (14)	0.0021 (10)	0.0016 (10)	0.0050 (11)
C4A	0.0250 (13)	0.0368 (14)	0.0369 (14)	0.0040 (10)	0.0014 (10)	0.0034 (11)
C5	0.0284 (13)	0.0405 (15)	0.0404 (14)	0.0057 (11)	0.0025 (11)	0.0034 (12)
C6	0.0384 (15)	0.0401 (15)	0.0400 (15)	0.0085 (12)	0.0013 (12)	0.0007 (12)
C7	0.0397 (15)	0.0359 (14)	0.0421 (15)	0.0002 (11)	−0.0026 (12)	−0.0011 (12)
C8	0.0274 (12)	0.0369 (14)	0.0452 (15)	−0.0001 (11)	−0.0006 (11)	−0.0018 (12)
C8A	0.0277 (13)	0.0332 (13)	0.0341 (14)	0.0024 (10)	0.0013 (10)	0.0025 (11)
C9	0.0217 (11)	0.0358 (14)	0.0383 (14)	−0.0013 (10)	0.0011 (10)	0.0000 (11)
C10	0.0222 (11)	0.0396 (15)	0.0383 (14)	−0.0004 (10)	0.0004 (10)	−0.0024 (12)
C11	0.0252 (12)	0.0343 (14)	0.0466 (15)	0.0003 (10)	−0.0023 (11)	−0.0021 (12)
C12	0.0397 (15)	0.0427 (16)	0.0439 (16)	−0.0075 (12)	−0.0004 (13)	−0.0037 (13)
C13	0.0394 (16)	0.0513 (18)	0.0415 (16)	−0.0040 (13)	0.0065 (12)	−0.0111 (14)
C14	0.0383 (15)	0.0447 (17)	0.0554 (18)	0.0001 (13)	0.0061 (13)	−0.0124 (15)
C15	0.0373 (15)	0.0397 (16)	0.0446 (16)	−0.0034 (12)	0.0027 (12)	−0.0015 (12)
C16	0.0233 (12)	0.0335 (14)	0.0500 (16)	−0.0038 (10)	−0.0020 (11)	−0.0011 (12)
C17	0.0217 (11)	0.0326 (13)	0.0467 (15)	−0.0025 (10)	−0.0028 (10)	−0.0032 (12)
C18	0.0339 (14)	0.0350 (14)	0.0471 (16)	0.0030 (11)	0.0001 (12)	−0.0014 (12)
C19	0.0380 (15)	0.0338 (14)	0.0499 (17)	0.0027 (11)	−0.0050 (13)	−0.0001 (12)
C20	0.0343 (15)	0.0474 (17)	0.0467 (16)	−0.0043 (12)	−0.0052 (12)	−0.0032 (13)
C21	0.0382 (16)	0.0508 (17)	0.0493 (18)	0.0058 (13)	−0.0048 (13)	−0.0154 (14)
C22	0.0365 (14)	0.0375 (15)	0.0524 (18)	0.0043 (12)	−0.0118 (12)	−0.0084 (13)

### 3-Benzyl-2-[(E)-2-(furan-2-yl)-1-methylvinyl]-2,3-dihydroquinazolin-4(1H)-one (II) Geometric parameters (Å, º)

**Table:**

O1—C14	1.364 (3)	C10—H10	0.9500
O1—C11	1.398 (3)	C11—C12	1.362 (4)
O2—C4	1.234 (3)	C12—C13	1.449 (4)
N1—C8A	1.386 (3)	C12—H12	0.9500
N1—C2	1.460 (3)	C13—C14	1.348 (4)
N1—H1	0.90 (3)	C13—H13	0.9500
C2—N3	1.493 (3)	C14—H14	0.9500
C2—C9	1.525 (3)	C15—H15A	0.9800
C2—H2	1.0000	C15—H15B	0.9800
N3—C4	1.378 (3)	C15—H15C	0.9800
N3—C16	1.483 (3)	C16—C17	1.520 (4)
C4—C4A	1.490 (4)	C16—H16A	0.9900
C4A—C8A	1.403 (3)	C16—H16B	0.9900
C4A—C5	1.404 (3)	C17—C22	1.390 (4)
C5—C6	1.395 (4)	C17—C18	1.398 (4)
C5—H5	0.9500	C18—C19	1.389 (4)
C6—C7	1.392 (4)	C18—H18	0.9500
C6—H6	0.9500	C19—C20	1.389 (4)
C7—C8	1.393 (4)	C19—H19	0.9500
C7—H7	0.9500	C20—C21	1.391 (4)
C8—C8A	1.408 (3)	C20—H20	0.9500
C8—H8	0.9500	C21—C22	1.395 (4)
C9—C10	1.351 (3)	C21—H21	0.9500
C9—C15	1.511 (4)	C22—H22	0.9500
C10—C11	1.455 (3)		
			
C14—O1—C11	107.0 (2)	C12—C11—C10	137.1 (2)
C8A—N1—C2	119.9 (2)	O1—C11—C10	113.8 (2)
C8A—N1—H1	117.1 (16)	C11—C12—C13	106.5 (2)
C2—N1—H1	111.2 (17)	C11—C12—H12	126.7
N1—C2—N3	109.3 (2)	C13—C12—H12	126.7
N1—C2—C9	108.9 (2)	C14—C13—C12	106.5 (2)
N3—C2—C9	111.59 (19)	C14—C13—H13	126.7
N1—C2—H2	109.0	C12—C13—H13	126.7
N3—C2—H2	109.0	C13—C14—O1	110.9 (3)
C9—C2—H2	109.0	C13—C14—H14	124.6
C4—N3—C16	117.6 (2)	O1—C14—H14	124.6
C4—N3—C2	122.2 (2)	C9—C15—H15A	109.5
C16—N3—C2	116.39 (19)	C9—C15—H15B	109.5
O2—C4—N3	121.9 (2)	H15A—C15—H15B	109.5
O2—C4—C4A	122.0 (2)	C9—C15—H15C	109.5
N3—C4—C4A	116.0 (2)	H15A—C15—H15C	109.5
C8A—C4A—C5	119.9 (2)	H15B—C15—H15C	109.5
C8A—C4A—C4	120.3 (2)	N3—C16—C17	112.9 (2)
C5—C4A—C4	119.5 (2)	N3—C16—H16A	109.0
C6—C5—C4A	120.7 (2)	C17—C16—H16A	109.0
C6—C5—H5	119.7	N3—C16—H16B	109.0
C4A—C5—H5	119.7	C17—C16—H16B	109.0
C7—C6—C5	119.1 (2)	H16A—C16—H16B	107.8
C7—C6—H6	120.4	C22—C17—C18	118.2 (2)
C5—C6—H6	120.4	C22—C17—C16	121.2 (2)
C6—C7—C8	121.2 (2)	C18—C17—C16	120.5 (2)
C6—C7—H7	119.4	C19—C18—C17	120.6 (2)
C8—C7—H7	119.4	C19—C18—H18	119.7
C7—C8—C8A	119.9 (2)	C17—C18—H18	119.7
C7—C8—H8	120.1	C18—C19—C20	120.6 (3)
C8A—C8—H8	120.1	C18—C19—H19	119.7
N1—C8A—C4A	119.2 (2)	C20—C19—H19	119.7
N1—C8A—C8	121.5 (2)	C19—C20—C21	119.3 (3)
C4A—C8A—C8	119.2 (2)	C19—C20—H20	120.4
C10—C9—C15	124.5 (2)	C21—C20—H20	120.4
C10—C9—C2	118.0 (2)	C20—C21—C22	119.8 (3)
C15—C9—C2	117.5 (2)	C20—C21—H21	120.1
C9—C10—C11	127.3 (2)	C22—C21—H21	120.1
C9—C10—H10	116.3	C17—C22—C21	121.4 (3)
C11—C10—H10	116.3	C17—C22—H22	119.3
C12—C11—O1	109.1 (2)	C21—C22—H22	119.3
			
C8A—N1—C2—N3	41.3 (3)	N1—C2—C9—C10	131.6 (2)
C8A—N1—C2—C9	163.4 (2)	N3—C2—C9—C10	−107.7 (2)
N1—C2—N3—C4	−36.7 (3)	N1—C2—C9—C15	−48.3 (3)
C9—C2—N3—C4	−157.3 (2)	N3—C2—C9—C15	72.5 (3)
N1—C2—N3—C16	165.83 (19)	C15—C9—C10—C11	−1.3 (4)
C9—C2—N3—C16	45.3 (3)	C2—C9—C10—C11	178.8 (2)
C16—N3—C4—O2	−11.4 (3)	C14—O1—C11—C12	0.3 (3)
C2—N3—C4—O2	−168.5 (2)	C14—O1—C11—C10	179.6 (2)
C16—N3—C4—C4A	171.8 (2)	C9—C10—C11—C12	−9.7 (5)
C2—N3—C4—C4A	14.6 (3)	C9—C10—C11—O1	171.3 (2)
O2—C4—C4A—C8A	−170.6 (2)	O1—C11—C12—C13	−0.1 (3)
N3—C4—C4A—C8A	6.2 (3)	C10—C11—C12—C13	−179.2 (3)
O2—C4—C4A—C5	4.5 (4)	C11—C12—C13—C14	−0.1 (3)
N3—C4—C4A—C5	−178.7 (2)	C12—C13—C14—O1	0.3 (3)
C8A—C4A—C5—C6	1.1 (4)	C11—O1—C14—C13	−0.4 (3)
C4—C4A—C5—C6	−174.0 (2)	C4—N3—C16—C17	106.2 (2)
C4A—C5—C6—C7	−0.5 (4)	C2—N3—C16—C17	−95.3 (2)
C5—C6—C7—C8	−0.5 (4)	N3—C16—C17—C22	120.2 (3)
C6—C7—C8—C8A	0.9 (4)	N3—C16—C17—C18	−58.8 (3)
C2—N1—C8A—C4A	−24.3 (3)	C22—C17—C18—C19	−1.4 (4)
C2—N1—C8A—C8	159.8 (2)	C16—C17—C18—C19	177.7 (2)
C5—C4A—C8A—N1	−176.6 (2)	C17—C18—C19—C20	−0.4 (4)
C4—C4A—C8A—N1	−1.5 (4)	C18—C19—C20—C21	1.6 (4)
C5—C4A—C8A—C8	−0.7 (4)	C19—C20—C21—C22	−1.2 (4)
C4—C4A—C8A—C8	174.4 (2)	C18—C17—C22—C21	1.8 (4)
C7—C8—C8A—N1	175.5 (2)	C16—C17—C22—C21	−177.2 (2)
C7—C8—C8A—C4A	−0.3 (4)	C20—C21—C22—C17	−0.6 (4)

### 3-Benzyl-2-[(E)-2-(furan-2-yl)-1-methylvinyl]-2,3-dihydroquinazolin-4(1H)-one (II) Hydrogen-bond geometry (Å, º)

**Table:**

*D*—H···*A*	*D*—H	H···*A*	*D*···*A*	*D*—H···*A*
N1—H1···O2^i^	0.90 (3)	2.07 (3)	2.971 (3)	174 (2)

Symmetry code: (i) *x*+1/2, −*y*+3/2, −*z*+1.

### 3-(Furan-2-ylmethyl)-2-[(E)-2-(furan-2-yl)-1-methylvinyl]-2,3-dihydroquinazolin-4(1H)-one (III) Crystal data

**Table:**

C_20_H_18_N_2_O_3_	*D*_x_ = 1.334 Mg m^−^^3^
*M**_r_* = 334.36	Synchrotron radiation, λ = 0.96990 Å
Orthorhombic, *P**b**c**a*	Cell parameters from 600 reflections
*a* = 13.928 (3) Å	θ = 3.2–32.0°
*b* = 10.684 (2) Å	µ = 0.19 mm^−^^1^
*c* = 22.368 (5) Å	*T* = 100 K
*V* = 3328.5 (12) Å^3^	Plate, yellow
*Z* = 8	0.30 × 0.30 × 0.07 mm
*F*(000) = 1408	

### 3-(Furan-2-ylmethyl)-2-[(E)-2-(furan-2-yl)-1-methylvinyl]-2,3-dihydroquinazolin-4(1H)-one (III) Data collection

**Table:**

Rayonix SX165 CCD diffractometer	2414 reflections with *I* > 2σ(*I*)
/f scan	*R*_int_ = 0.097
Absorption correction: multi-scan (*SCALA*; Evans, 2006)	θ_max_ = 38.4°, θ_min_ = 3.2°
*T*_min_ = 0.940, *T*_max_ = 0.980	*h* = −17→17
27461 measured reflections	*k* = −13→13
3460 independent reflections	*l* = −26→26

### 3-(Furan-2-ylmethyl)-2-[(E)-2-(furan-2-yl)-1-methylvinyl]-2,3-dihydroquinazolin-4(1H)-one (III) Refinement

**Table:**

Refinement on *F*^2^	Secondary atom site location: difference Fourier map
Least-squares matrix: full	Hydrogen site location: mixed
*R*[*F*^2^ > 2σ(*F*^2^)] = 0.089	H atoms treated by a mixture of independent and constrained refinement
*wR*(*F*^2^) = 0.224	*w* = 1/[σ^2^(*F*_o_^2^) + (0.05*P*)^2^ + 6*P*] where *P* = (*F*_o_^2^ + 2*F*_c_^2^)/3
*S* = 1.05	(Δ/σ)_max_ < 0.001
3460 reflections	Δρ_max_ = 0.42 e Å^−^^3^
231 parameters	Δρ_min_ = −0.57 e Å^−^^3^
0 restraints	Extinction correction: SHELXL, Fc^*^=kFc[1+0.001xFc^2^λ^3^/sin(2θ)]^-1/4^
Primary atom site location: difference Fourier map	Extinction coefficient: 0.0078 (7)

### 3-(Furan-2-ylmethyl)-2-[(E)-2-(furan-2-yl)-1-methylvinyl]-2,3-dihydroquinazolin-4(1H)-one (III) Special details

**Table:**

Geometry. All esds (except the esd in the dihedral angle between two l.s. planes) are estimated using the full covariance matrix. The cell esds are taken into account individually in the estimation of esds in distances, angles and torsion angles; correlations between esds in cell parameters are only used when they are defined by crystal symmetry. An approximate (isotropic) treatment of cell esds is used for estimating esds involving l.s. planes.

### 3-(Furan-2-ylmethyl)-2-[(E)-2-(furan-2-yl)-1-methylvinyl]-2,3-dihydroquinazolin-4(1H)-one (III) Fractional atomic coordinates and isotropic or equivalent isotropic displacement parameters (Å^2^)

**Table:**

	*x*	*y*	*z*	*U*_iso_*/*U*_eq_	
O1	0.53713 (18)	0.6665 (2)	0.63004 (12)	0.0493 (7)	
O2	0.1753 (2)	0.2666 (3)	0.64505 (16)	0.0672 (9)	
O3	0.12657 (16)	0.2418 (3)	0.49520 (14)	0.0646 (9)	
N1	0.41678 (19)	0.2216 (3)	0.50272 (13)	0.0392 (7)	
H1	0.483 (3)	0.224 (3)	0.5002 (16)	0.047*	
C2	0.3738 (2)	0.3381 (3)	0.52287 (15)	0.0390 (8)	
H2	0.3852	0.4045	0.4922	0.047*	
N3	0.26847 (17)	0.3192 (3)	0.53043 (13)	0.0432 (8)	
C4	0.2161 (2)	0.2432 (4)	0.49362 (18)	0.0475 (10)	
C4A	0.2709 (2)	0.1577 (3)	0.45443 (16)	0.0434 (9)	
C5	0.2225 (3)	0.0789 (4)	0.41411 (19)	0.0551 (11)	
H5	0.1550	0.0868	0.4093	0.066*	
C6	0.2718 (3)	−0.0095 (4)	0.3815 (2)	0.0597 (12)	
H6	0.2387	−0.0630	0.3546	0.072*	
C7	0.3707 (3)	−0.0196 (4)	0.38855 (19)	0.0537 (10)	
H7	0.4049	−0.0808	0.3663	0.064*	
C8	0.4203 (3)	0.0584 (3)	0.42746 (17)	0.0461 (9)	
H8	0.4879	0.0506	0.4314	0.055*	
C8A	0.3708 (2)	0.1484 (3)	0.46093 (15)	0.0390 (8)	
C9	0.4206 (2)	0.3773 (3)	0.58156 (15)	0.0346 (7)	
C10	0.4528 (2)	0.4952 (3)	0.58733 (15)	0.0357 (7)	
H10	0.4455	0.5483	0.5536	0.043*	
C11	0.4974 (2)	0.5494 (3)	0.63964 (15)	0.0374 (8)	
C12	0.5116 (3)	0.5158 (3)	0.69790 (16)	0.0477 (9)	
H12	0.4917	0.4401	0.7164	0.057*	
C13	0.5625 (3)	0.6176 (4)	0.72574 (19)	0.0569 (11)	
H13	0.5826	0.6224	0.7663	0.068*	
C14	0.5760 (3)	0.7043 (4)	0.68329 (19)	0.0533 (10)	
H14	0.6083	0.7816	0.6893	0.064*	
C15	0.4279 (2)	0.2790 (3)	0.62950 (16)	0.0388 (8)	
H15A	0.4948	0.2721	0.6428	0.058*	
H15B	0.4065	0.1983	0.6135	0.058*	
H15C	0.3873	0.3026	0.6635	0.058*	
C16	0.2168 (2)	0.4154 (4)	0.56476 (18)	0.0523 (10)	
H16A	0.1519	0.4261	0.5476	0.063*	
H16B	0.2512	0.4961	0.5607	0.063*	
C17	0.2076 (2)	0.3842 (3)	0.62962 (17)	0.0402 (8)	
C18	0.2340 (3)	0.4544 (3)	0.67787 (16)	0.0546 (11)	
H18	0.2623	0.5353	0.6783	0.066*	
C19	0.2087 (4)	0.3767 (5)	0.7279 (2)	0.0819 (17)	
H19	0.2137	0.4003	0.7687	0.098*	
C20	0.1777 (3)	0.2682 (5)	0.7084 (3)	0.0787 (17)	
H20	0.1593	0.2003	0.7333	0.094*	

### 3-(Furan-2-ylmethyl)-2-[(E)-2-(furan-2-yl)-1-methylvinyl]-2,3-dihydroquinazolin-4(1H)-one (III) Atomic displacement parameters (Å^2^)

**Table:**

	*U*^11^	*U*^22^	*U*^33^	*U*^12^	*U*^13^	*U*^23^
O1	0.0430 (14)	0.0457 (14)	0.0591 (17)	−0.0026 (11)	0.0040 (12)	0.0069 (12)
O2	0.0530 (17)	0.0562 (17)	0.092 (2)	−0.0120 (13)	0.0018 (16)	0.0252 (16)
O3	0.0191 (12)	0.0779 (19)	0.097 (2)	−0.0025 (11)	−0.0024 (13)	0.0442 (16)
N1	0.0194 (13)	0.0550 (18)	0.0431 (17)	0.0007 (12)	−0.0018 (11)	−0.0013 (13)
C2	0.0246 (16)	0.053 (2)	0.0396 (19)	0.0017 (14)	0.0008 (13)	0.0122 (15)
N3	0.0180 (13)	0.0623 (18)	0.0494 (18)	0.0049 (12)	0.0032 (12)	0.0202 (15)
C4	0.0201 (15)	0.064 (2)	0.059 (2)	−0.0056 (15)	−0.0048 (15)	0.0344 (19)
C4A	0.0258 (16)	0.055 (2)	0.050 (2)	−0.0099 (15)	−0.0069 (15)	0.0240 (17)
C5	0.040 (2)	0.056 (2)	0.069 (3)	−0.0214 (18)	−0.0226 (19)	0.033 (2)
C6	0.055 (2)	0.047 (2)	0.077 (3)	−0.0234 (19)	−0.027 (2)	0.019 (2)
C7	0.051 (2)	0.046 (2)	0.064 (3)	−0.0138 (17)	−0.0180 (19)	0.0059 (18)
C8	0.0353 (18)	0.049 (2)	0.053 (2)	−0.0092 (15)	−0.0078 (16)	0.0025 (17)
C8A	0.0268 (16)	0.0492 (19)	0.0411 (19)	−0.0095 (14)	−0.0055 (14)	0.0128 (15)
C9	0.0256 (15)	0.0421 (17)	0.0362 (18)	0.0072 (13)	0.0037 (12)	0.0089 (14)
C10	0.0281 (16)	0.0392 (17)	0.0398 (18)	0.0057 (13)	0.0058 (13)	0.0060 (14)
C11	0.0331 (17)	0.0330 (15)	0.046 (2)	0.0089 (13)	0.0101 (14)	0.0050 (14)
C12	0.064 (2)	0.0369 (18)	0.042 (2)	0.0108 (17)	0.0086 (18)	−0.0027 (15)
C13	0.076 (3)	0.047 (2)	0.048 (2)	0.015 (2)	0.005 (2)	−0.0113 (18)
C14	0.051 (2)	0.047 (2)	0.063 (3)	0.0034 (17)	0.0029 (19)	−0.0081 (19)
C15	0.0380 (18)	0.0364 (17)	0.042 (2)	0.0068 (13)	0.0013 (15)	0.0079 (14)
C16	0.0288 (17)	0.058 (2)	0.070 (3)	0.0146 (16)	0.0111 (17)	0.028 (2)
C17	0.0269 (15)	0.0302 (16)	0.064 (2)	0.0049 (12)	0.0127 (15)	0.0106 (15)
C18	0.098 (3)	0.0220 (15)	0.044 (2)	0.0102 (18)	0.025 (2)	−0.0029 (15)
C19	0.127 (5)	0.070 (3)	0.049 (3)	0.047 (3)	0.032 (3)	0.002 (2)
C20	0.068 (3)	0.067 (3)	0.102 (4)	0.020 (2)	0.046 (3)	0.043 (3)

### 3-(Furan-2-ylmethyl)-2-[(E)-2-(furan-2-yl)-1-methylvinyl]-2,3-dihydroquinazolin-4(1H)-one (III) Geometric parameters (Å, º)

**Table:**

O1—C14	1.369 (5)	C8—H8	0.9500
O1—C11	1.386 (4)	C9—C10	1.342 (5)
O2—C17	1.378 (4)	C9—C15	1.504 (4)
O2—C20	1.418 (6)	C10—C11	1.446 (5)
O3—C4	1.247 (4)	C10—H10	0.9500
N1—C8A	1.377 (4)	C11—C12	1.366 (5)
N1—C2	1.453 (4)	C12—C13	1.441 (6)
N1—H1	0.92 (4)	C12—H12	0.9500
C2—N3	1.491 (4)	C13—C14	1.339 (6)
C2—C9	1.524 (5)	C13—H13	0.9500
C2—H2	1.0000	C14—H14	0.9500
N3—C4	1.367 (5)	C15—H15A	0.9800
N3—C16	1.471 (5)	C15—H15B	0.9800
C4—C4A	1.478 (6)	C15—H15C	0.9800
C4A—C8A	1.402 (4)	C16—C17	1.494 (5)
C4A—C5	1.406 (5)	C16—H16A	0.9900
C5—C6	1.376 (6)	C16—H16B	0.9900
C5—H5	0.9500	C17—C18	1.365 (5)
C6—C7	1.390 (6)	C18—C19	1.437 (6)
C6—H6	0.9500	C18—H18	0.9500
C7—C8	1.389 (5)	C19—C20	1.311 (7)
C7—H7	0.9500	C19—H19	0.9500
C8—C8A	1.400 (5)	C20—H20	0.9500
			
C14—O1—C11	106.8 (3)	C9—C10—H10	116.7
C17—O2—C20	103.4 (3)	C11—C10—H10	116.7
C8A—N1—C2	120.4 (3)	C12—C11—O1	109.1 (3)
C8A—N1—H1	116 (2)	C12—C11—C10	136.8 (3)
C2—N1—H1	114 (2)	O1—C11—C10	114.1 (3)
N1—C2—N3	108.9 (3)	C11—C12—C13	106.6 (3)
N1—C2—C9	109.1 (3)	C11—C12—H12	126.7
N3—C2—C9	111.1 (3)	C13—C12—H12	126.7
N1—C2—H2	109.2	C14—C13—C12	106.5 (4)
N3—C2—H2	109.2	C14—C13—H13	126.7
C9—C2—H2	109.2	C12—C13—H13	126.7
C4—N3—C16	117.9 (3)	C13—C14—O1	111.0 (4)
C4—N3—C2	122.5 (3)	C13—C14—H14	124.5
C16—N3—C2	116.4 (3)	O1—C14—H14	124.5
O3—C4—N3	121.6 (4)	C9—C15—H15A	109.5
O3—C4—C4A	121.7 (4)	C9—C15—H15B	109.5
N3—C4—C4A	116.6 (3)	H15A—C15—H15B	109.5
C8A—C4A—C5	120.0 (4)	C9—C15—H15C	109.5
C8A—C4A—C4	119.7 (3)	H15A—C15—H15C	109.5
C5—C4A—C4	120.2 (3)	H15B—C15—H15C	109.5
C6—C5—C4A	120.8 (3)	N3—C16—C17	113.1 (3)
C6—C5—H5	119.6	N3—C16—H16A	109.0
C4A—C5—H5	119.6	C17—C16—H16A	109.0
C5—C6—C7	119.2 (4)	N3—C16—H16B	109.0
C5—C6—H6	120.4	C17—C16—H16B	109.0
C7—C6—H6	120.4	H16A—C16—H16B	107.8
C8—C7—C6	121.1 (4)	C18—C17—O2	113.0 (3)
C8—C7—H7	119.4	C18—C17—C16	128.5 (3)
C6—C7—H7	119.4	O2—C17—C16	118.3 (3)
C7—C8—C8A	120.2 (3)	C17—C18—C19	103.4 (4)
C7—C8—H8	119.9	C17—C18—H18	128.3
C8A—C8—H8	119.9	C19—C18—H18	128.3
N1—C8A—C8	121.6 (3)	C20—C19—C18	109.5 (4)
N1—C8A—C4A	119.5 (3)	C20—C19—H19	125.3
C8—C8A—C4A	118.8 (3)	C18—C19—H19	125.3
C10—C9—C15	124.3 (3)	C19—C20—O2	110.5 (4)
C10—C9—C2	118.9 (3)	C19—C20—H20	124.8
C15—C9—C2	116.8 (3)	O2—C20—H20	124.8
C9—C10—C11	126.7 (3)		
			
C8A—N1—C2—N3	40.4 (4)	N1—C2—C9—C10	130.5 (3)
C8A—N1—C2—C9	161.8 (3)	N3—C2—C9—C10	−109.4 (3)
N1—C2—N3—C4	−35.4 (4)	N1—C2—C9—C15	−49.0 (4)
C9—C2—N3—C4	−155.6 (3)	N3—C2—C9—C15	71.0 (4)
N1—C2—N3—C16	164.9 (3)	C15—C9—C10—C11	−1.5 (5)
C9—C2—N3—C16	44.8 (4)	C2—C9—C10—C11	179.0 (3)
C16—N3—C4—O3	−10.5 (5)	C14—O1—C11—C12	0.1 (4)
C2—N3—C4—O3	−169.9 (3)	C14—O1—C11—C10	−180.0 (3)
C16—N3—C4—C4A	173.0 (3)	C9—C10—C11—C12	−9.7 (6)
C2—N3—C4—C4A	13.6 (4)	C9—C10—C11—O1	170.4 (3)
O3—C4—C4A—C8A	−169.9 (3)	O1—C11—C12—C13	0.1 (4)
N3—C4—C4A—C8A	6.7 (4)	C10—C11—C12—C13	−179.8 (4)
O3—C4—C4A—C5	5.2 (5)	C11—C12—C13—C14	−0.3 (4)
N3—C4—C4A—C5	−178.3 (3)	C12—C13—C14—O1	0.4 (4)
C8A—C4A—C5—C6	1.3 (5)	C11—O1—C14—C13	−0.3 (4)
C4—C4A—C5—C6	−173.7 (3)	C4—N3—C16—C17	105.9 (4)
C4A—C5—C6—C7	−0.5 (6)	C2—N3—C16—C17	−93.5 (4)
C5—C6—C7—C8	−0.4 (6)	C20—O2—C17—C18	3.2 (4)
C6—C7—C8—C8A	0.6 (6)	C20—O2—C17—C16	178.6 (3)
C2—N1—C8A—C8	160.4 (3)	N3—C16—C17—C18	125.8 (4)
C2—N1—C8A—C4A	−24.1 (5)	N3—C16—C17—O2	−48.8 (4)
C7—C8—C8A—N1	175.7 (3)	O2—C17—C18—C19	−4.7 (4)
C7—C8—C8A—C4A	0.2 (5)	C16—C17—C18—C19	−179.5 (4)
C5—C4A—C8A—N1	−176.7 (3)	C17—C18—C19—C20	4.5 (5)
C4—C4A—C8A—N1	−1.7 (5)	C18—C19—C20—O2	−2.8 (6)
C5—C4A—C8A—C8	−1.1 (5)	C17—O2—C20—C19	−0.1 (5)
C4—C4A—C8A—C8	173.9 (3)		

### 3-(Furan-2-ylmethyl)-2-[(E)-2-(furan-2-yl)-1-methylvinyl]-2,3-dihydroquinazolin-4(1H)-one (III) Hydrogen-bond geometry (Å, º)

**Table:**

*D*—H···*A*	*D*—H	H···*A*	*D*···*A*	*D*—H···*A*
N1—H1···O3^i^	0.92 (4)	2.04 (4)	2.949 (4)	169 (3)

Symmetry code: (i) *x*+1/2, −*y*+1/2, −*z*+1.
